# External gap progression after cyclic fatigue of adhesive overlays and crowns made with high translucency zirconia or lithium silicate

**DOI:** 10.1111/jerd.12837

**Published:** 2021-11-16

**Authors:** Andrea Baldi, Allegra Comba, Giorgio Ferrero, Edoardo Italia, Riccardo Michelotto Tempesta, Gaetano Paolone, Annalisa Mazzoni, Lorenzo Breschi, Nicola Scotti

**Affiliations:** ^1^ Department of Surgical Sciences Dental School Lingotto Turin; ^2^ Department of Dentistry, IRCCS San Raffaele Hospital and Dental School Vita Salute University Milan Italy; ^3^ Department of Biomedical and Neuromotor Sciences, DIBINEM University of Bologna, Alma Mater Studiorum Bologna Italy

**Keywords:** crown, endodontically treated teeth, external gap, high‐translucency zirconia, micro‐CT, overlay

## Abstract

**Objectives:**

To evaluate three‐dimensional external gap progression after chewing simulation of high translucency zirconia (HTZ) and zirconia‐reinforced lithium silicate (ZLS) applied on endodontically treated teeth with different preparation designs.

**Materials and Method:**

Endodontically treated molars were prepared with low‐retentive (adhesive overlay) and high‐retentive (full crown) designs above cementum‐enamel junction and restored with HTZ and ZLS. Micro‐computed tomography analysis was assessed before and after chewing simulation to evaluate three‐dimensionally the external gap progression. Results were statistically analyzed with two‐way ANOVA and post‐hoc Tukey test.

**Results:**

High‐retentive preparation design had a significantly inferior gap progression compared to the overlay preparation (*p* < 0.01); ZLS exhibited a significant inferior gap progression compared to HTZ (*p* < 0.01).

**Conclusions:**

High‐retentive preparations restored with ZLS seem to better perform in maintaining the sealing of the external margin after cyclic fatigue.

**Clinical significance:**

The clinician should pay attention to the proper combination of preparation designs and ceramic material selection for an endodontically treated molar restoration. HTZ seems to perform worse than lithium silicate in terms of marginal sealing, still showing lacks in resistance to cyclic fatigue when adhesive preparations are performed.

## 1 INTRODUCTION

Modern restorative procedures on endodontically treated teeth (ETT) aim to improve their mechanical properties, which are inferior to those of their vital counterparts,^1^, ^2^ while being minimally invasive to healthy dental tissues. To accomplish these goals, ETT are frequently restored with adhesive procedures and partial restorations which represent a valid alternative to conventional crowns.^3^, ^4^, ^5^

Several materials have been successfully applied in full‐coverage adhesive restorations on ETT, such as glass‐reinforced ceramics, resin composites, and hybrid materials.^6^, ^7^, ^8^ These materials showed good performance in both in vitro and in vivo studies.^9^, ^10^ However, every year, new restorative materials are developed and produced with the aim of restoring the optical and mechanical properties of natural teeth, even in severely compromised teeth.

Among the recently introduced monolithic CAD/CAM materials that can be used for cuspal coverage indirect restorations on severely damaged teeth, zirconia has certainly experienced the greatest evolution. In particular, high translucency zirconia (HTZ) has been recently introduced in restorative dentistry, replacing the tetragonal version, especially for monolithic single‐tooth restorations. The introduction of a variable amount of cubic phase, which is optically isotropic, was meant to improve the translucency of the material, at the expense of strength and toughness due to the lack of transformation toughening and the coarser microstructure.^11^ As a recent study pointed out, cubic grains are wider than tetragonal ones and generate more stabilizing oxides, making the tetragonal phase more prone to aging.^12^ As result, HTZ was initially considered less suitable for posterior restorations and indicated only for the anterior area. Today, however, industries have been able to produce various types of zirconia with varying percentages of cubic phase, ultimately creating HTZ specifically indicated for the posterior sectors and with a good balance between optical and mechanical proprieties.^13^ On the other hand, an alternative ceramic material with high mechanical and esthetic performances suitable for cuspal coverage restorations is the zirconia‐reinforced lithium silicate ceramics (ZLS). Its microstructure has a homogeneous glassy matrix which contains a crystalline component made of round and submicrometric elongated grains of lithium metasilicates and lithium orthophosphates; in addition to these, tetragonal zirconia fillers are added, aimed at increasing strength values, obtain favorable optical properties within increased mechanical characteristics compared to other glass‐ceramics.^14^, ^15^

A crucial consideration when dealing with adhesive preparations is the luting protocol and its efficiency, since the adhesive preparation design is, by definition, less macromechanically retentive than a conventional crown. Despite significant developments in adhesive protocols towards enamel and dentin, failures related to secondary caries are still the major issue when adhesive restorations are addressed,^9^, ^10^ above all with unexperienced operators.^16^ It should be considered that, prior to clinical dramatic failure, usually considered as the restoration debonding or fracture, the interfacial gap formation plays an important role as it represent the first sign of restoration deterioration, since this hard‐to‐clean area contribute to the reduction of the tooth‐restoration complex's resistance^5^, ^17^ and it can lead to bacterial recolonization of the tooth crown and the root canal system, with subsequent endodontic failure.^18^ These interfacial gaps tend to progressively expand during oral function and parafunction due to fatigue stresses from cyclic loading.^19^, ^20^, ^21^ Therefore, as highlighted in a recent review, fatigue parameters obtained from cyclic loading experiments should be considered more reliable predictors of the mechanical performance of contemporary adhesive restorative materials than quasi‐static mechanical properties.^22^ Moreover, the scientific community has put forth significant effort in testing and proposing adhesive treatments able to ensure effective bonding and interfacial seals using HTZ^23^ to let the material be employable in low‐retentive minimally invasive preparations. The absence of a glassy phase makes the bonding mechanisms of HTZ to dental tissues more difficult^24^: recent studies showed how the physicochemical conditioning method tends to increase the bond strength of resin‐based cements towards zirconia.^23^, ^25^ However, to the best of our knowledge, no studies reported the effects of fatigue cycling on the external gap opening of ETT restored with indirect adhesive restorations made with HTZ or ZLS.

The aim of the present in vitro study was to evaluate the external gap progression after cyclic fatigue of HTZ and ZLS applied on ETT with low and high retentive preparation designs. The following null hypotheses were tested: (1) there is no difference in terms of external gap progression between low‐retentive and high‐retentive preparation designs, and (2) there is no difference between HTZ and ZLS.

## 2 MATERIALS AND METHODS

### 2.1 Study design

This study was designed in four study groups (*n* = 12 each), where the specimens were randomly allocated considering:

- “Preparation design” in two levels: extracted molars, once endodontically treated, were prepared for a cuspal coverage restoration with two different designs: a low‐retentive adhesive overlay preparation and a high‐retentive full crown preparation with margin located 1 mm above cementum‐enamel junction (CEJ).
- “Restorative material” in two levels: Cuspal coverage adhesive restorations were performed using two different cad‐cam monolithic materials: a HTZ designed for posterior teeth (Katana STML, Kuraray Noritake) and a ZLS (Celtra Duo, Dentsply).

The materials employed in the present study are detailed in Table 1.

**TABLE 1:** General description of the main materials used in the present study

	Description	Manufacturer	Composition
KATANA STML	High translucency zirconia	Kuraray Noritake	Zirconium oxide (wt%: 59.9% c‐ZrO_2_, 39.5% t‐ZrO_2_, 0.4% m‐ZrO_2_, 0.2% r‐ZrO_2_), 4.8% Y_2_O_3_, pigments
Celtra Duo	Zirconia‐reinforced lithium silicate	Dentsply	58% Silicon dioxide, 10.1% crystallized zirconium dioxide, 10% zirconium dioxide, 5% phosphorous pentoxide, 2.0% ceria, 1.9% alumina, 1% terbium oxide
CLEARFIL MAJESTY ES‐2	Nanohybrid resin composite	Kuraray Noritake	Bisphenol A diglycidyl methacrylate, barium glass, pre‐polymerized organic filler, hydrophobic aromatic dimethacrylate, hydrophobic aliphatic dimethacrylate dl‐Camphorquinone, accelerators, initiators, pigments
PANAVIA V5	Dual resin cement	Kuraray Noritake	Bis‐GMA, TEGDMA, aromatic and aliphatic multifunctional monomer, accelerators, dl‐Camphorquinone, surface‐treated barium glass, fluoroaluminosilicate glass, fine particulate
CLEARFIL SE BOND 2	Two‐bottle self‐etch adhesive	Kuraray Noritake	Primer: 10‐MDP, HEMA, hydrophilic dimethacrylate, photoinitiator, water Bond: 10‐MDP, dimethacrylate resins, HEMA, Vitrebond copolymer, ethanol, water, filler, initiators, silane

### 2.2 Sample preparation

A total of 48 (*n* = 48) human sound upper molars were selected for the present study within 2 months from extraction due to periodontal reason. The inclusion criteria were as follow: sound teeth, similar root (length > 12 mm) and crown size (10 mm ± 2 mesiodistal, 10 mm ± 2 bucco‐oral) and no crack or demineralization under visual examination with light trans‐illumination and magnification. After proper disinfection (ultrasonic scaling and 0.5% chloramine for 48 h), selected teeth were stored in distilled water at 37°C.

Each specimen was endodontically treated by the same operator using PathFiles (1–2–3) and ProTaper Next (X1/X2) (Dentsply Maillefer) to reach the working length, set at the visible apical foramen. Irrigation was performed with 5% NaOCl (Niclor 5, OGNA) alternated with 10% EDTA (Tubuliclean, OGNA). Thereafter, specimens were obturated with gutta‐percha points (Gutta‐Percha Points Medium, Inline, B&M Dental) using a Down Pack heat source (Hu‐Friedy) and an endodontic sealer (Pulp Canal Sealer EWT, Kerr). Backfilling was performed with the Obtura III system (Analytic Technologies).

A standardized mesio‐occlusal‐distal cavity was prepared by an expert operator setting the residual wall thickness of the buccal and oral cusps at the height of the contour to 1.5 ± 0.2 mm, measured with a conventional caliper. Mesial and distal boxes were finished with dedicated sonic points (n°34 and 35, SONICflex, KaVo) to standardize their dimensions. A core composite build‐up was performed for all specimens, following the same protocol. A 30‐s selective enamel etching was performed with 35% phosphoric acid (K‐ETCHANT, Kuraray Noritake Dental), then rinsed for 30 s and air‐dried. Then, a self‐etch adhesive was applied (CLEARFIL SE BOND 2, Kuraray Noritake) following the manufacturer's instructions. Build‐up restoration was performed with a nanohybrid resin composite (CLEARFIL MAJESTY ES‐2, Kuraray Noritake) with a 2‐mm‐thick oblique layering technique. Light curing of both adhesive and resin composites was accomplished with an LED curing lamp (Celalux 2, VOCO) using a conventional program for 20 s at 1000 mW/cm^2^.

Samples were randomly allocated to one of two groups (*n* = 24 each) using https://www.randomizer.org/ according to the selected preparation design:

- Low‐retentive (ADH). A standardized 1.5 mm occlusal reduction was performed with a cylindrical bur (6836 KR 014, Komet) following occlusal anatomy. Boxes were finished with dedicated sonic points (n°34 and 35, SONICflex, KaVo) to remove eventual built‐up composite excesses. Finally, the occlusal margins were beveled with a football‐shaped bur (8368 L, Komet), and all corners were rounded with an Arkansas tip (661, Komet) and a rubber point (9436 M, Komet).
- High‐retentive (CRW). A standardized 1.5 mm full preparation was executed with a chamfer margin 1 ± 0.5 mm above CEJ. Both initial preparation and finishing were performed with dedicated chamfer burs (6881 014, Komet; 8881 014, Komet). Finally, all corners were rounded with an Arkansas tip (661, Komet) and a rubber point (9436 M, Komet).

**FIGURE 1:**
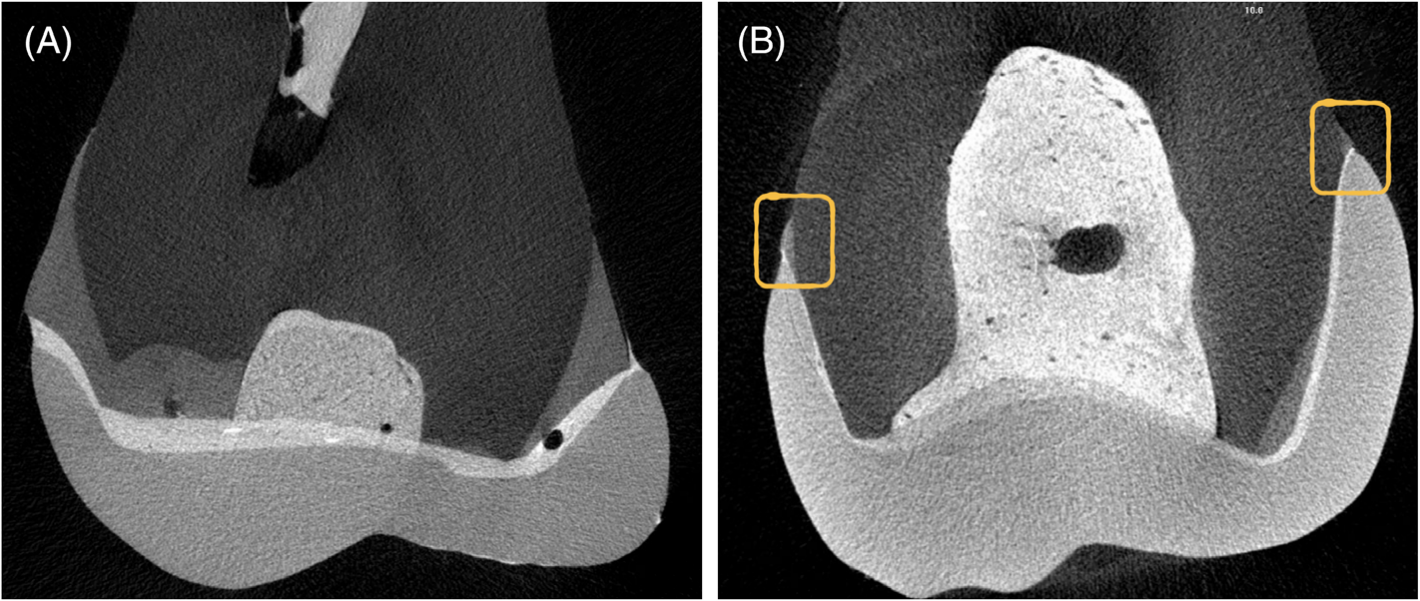
Random samples transversal sections of a low‐retentive design (Fig. 1A) and high‐retentive design (Fig. 1B). Both the restorations were performed above the CEJ level, as highlighted in Fig. 1B

An exemplificative image reporting transversal sections of a low‐retentive and high‐retentive designs is reported in Figure 1.

Samples were scanned with an intraoral scanner (CEREC Omnicam, Dentsply) and divided into two subgroups (*n* = 12 each) according to the CAD/CAM material employed: HTZ (KATANA, Kuraray Noritake) and ZLS (Celtra Duo, Dentsply). All restorations were designed with a CAD system, that allowed to standardize a minimum of 1.5 mm thickness (Cerec 4.5.2 software, Dentisply, Sirona, Konstanz, Germany) and milled with material‐specific default settings in extra‐fine mode (Cerec MC XL, Dentsply, Sirona, Konstanz, Germany). In all specimens, the parameters for luting space and minimum occlusal ceramic thickness were set to 80 μm and 1.5 mm, respectively. Once milled, ZLS was crystallized (Cerec Speedfire, Dentisply, Sirona, Konstanz, Germany) and HTZ was sintered according to the manufacturer instructions. Each restoration was luted with a dual‐cure resin cement, following the manufacturer's instructions (PANAVIA V5, Kuraray Noritake). Either ADH either CRW were cemented with digital pressure applied by the same operator, with more than 10 years of clinical experience, until fully seated onto the tooth margin. Table 2 reports the details of the adhesive procedures performed on both teeth and restorative materials. Excesses of cement were removed with a micro‐brush, then, after 3 min of setting, photopolymerization was carried out for a total of 3 min (approximately 40 s per surface) with an LED lamp at 1000 mW/cm^2^ (Celalux 2, VOCO). Finishing and polishing were performed with fine and extra‐fine diamond burs and rubber points on a handpiece. Margins were double‐checked to exclude samples with under‐contours, while over‐contours were corrected with a new cycle of finishing and polishing. All samples were confirmed to be clinically acceptable by an expert operator (more than 10 years of experience in prosthodontic field).

**TABLE 2:** Detailed adhesive procedures performed on different materials

Substrate	Adhesive procedure performed
Tooth	Enamel etching for 15 s, rinse and dry, apply tooth primer (PANAVIA V5 kit, Kuraray Noritake) for 20 s, dry with air
HTZ	Dry sandblasting with 50 micron alumina powder (RONDOflex Plus 360, KaVo), 5‐min ultrasonic bath in 98% alcohol, dry, apply CERAMIC PRIMER PLUS (PANAVIA V5 kit, Kuraray Noritake) for 20 s, dry, apply PANAVIA V5 cement through dedicated mixing tips
ZLS	9.6% Hydrofluoric acid (Porcelain Etch Gel, Pulpdent) for 30 s, 5‐min ultrasonic bath in 98% alcohol, dry, apply CERAMIC PRIMER PLUS (PANAVIA V5 kit, Kuraray Noritake) for 20 s, dry, apply PANAVIA V5 cement through dedicated mixing tips

### 2.3 Micro‐CT acquisition and fatigue simulation

Samples first underwent a micro‐CT scan (Skyscan 1172, Bruker) with the following parameters: voltage = 100 kV, current = 100 A, aluminum and copper (Al + Cu) filter, pixel size = 10 μm, averaging = 4, and rotation step = 0.1°. Images were reconstructed (NRecon, Bruker) to obtain DICOM files with standardized parameters: beam hardening correction = 15%, smoothing = 5, and ring artifact reduction = 6. HTZ samples (SG1) showed a higher radiopacity than ZLS, which was managed by doubling the aluminum and copper filters, using averaging = 7, and properly positioning the sample.

Fatigue simulation was accomplished with a chewing simulator (CS‐4.4, SD Mechatronik) using 6 mm diameter steatite balls as antagonists with the following settings: load = 50 N, frequency = 1 Hz, speed = 16 mm/s, sliding = 2 mm over the buccal triangular crest, and number of cycles = 500,000. A loading force of 50 N was selected in accordance with previous studies on fatigue testing.^26^, ^27^, ^28^

### 2.4 External gap progression analysis

**FIGURE 2:**
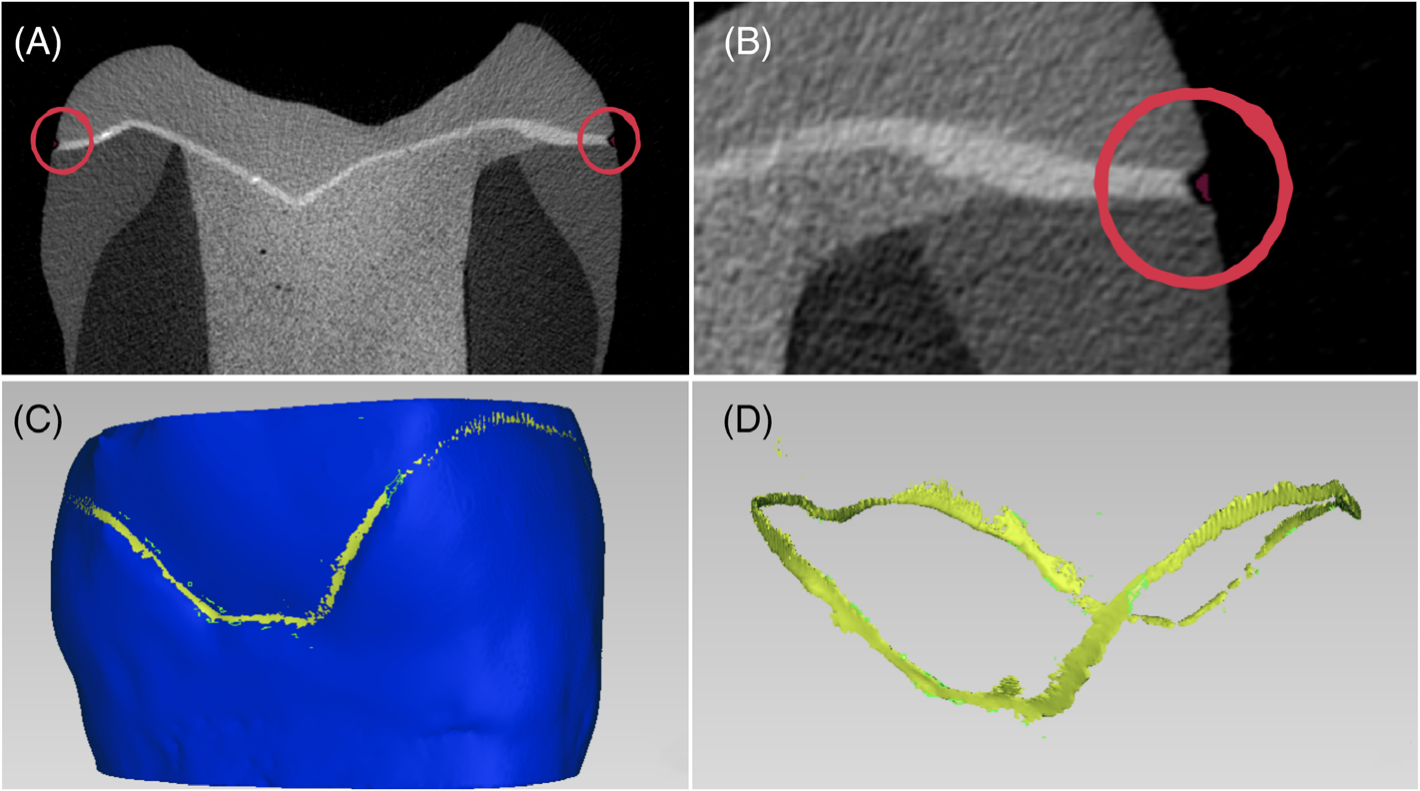
Random sample external gap analysis (ADH, ZLS) in stages A–D. Figure A presents a random cross‐section with external gaps highlighted. Figure B is a magnification of Figure A, showing in red the pixels corresponding to the external gap used in the analysis. Figure C shows a 3D rendering (Geomagic Studio 12, 3D Systems) of the tooth‐restoration complex (in blue) and the analyzed gap (in yellow). Figure D presents the analyzed gap in yellow

Specimens which survived to chewing simulation were submitted to a second micro‐CT scan, with the same parameters as the baseline, to maintain data consistency and evaluate the effect of fatigue on external gap progression. The obtained DICOM data were imported into a segmentation software (Mimics Medical 20.0, Materialise). A standardized workflow consisting of thresholding, region growing, and Boolean and morphological operations was used to specifically analyze the external interfacial gap. Segmented masks were converted into optimal quality STL files and imported into the analysis software (Geomagic Studio 12, 3D Systems) for noise removal and volume calculation (mm^3^). Figure 2A–D presents a schematic representation of the protocol steps for clarification.

**FIGURE 3:**
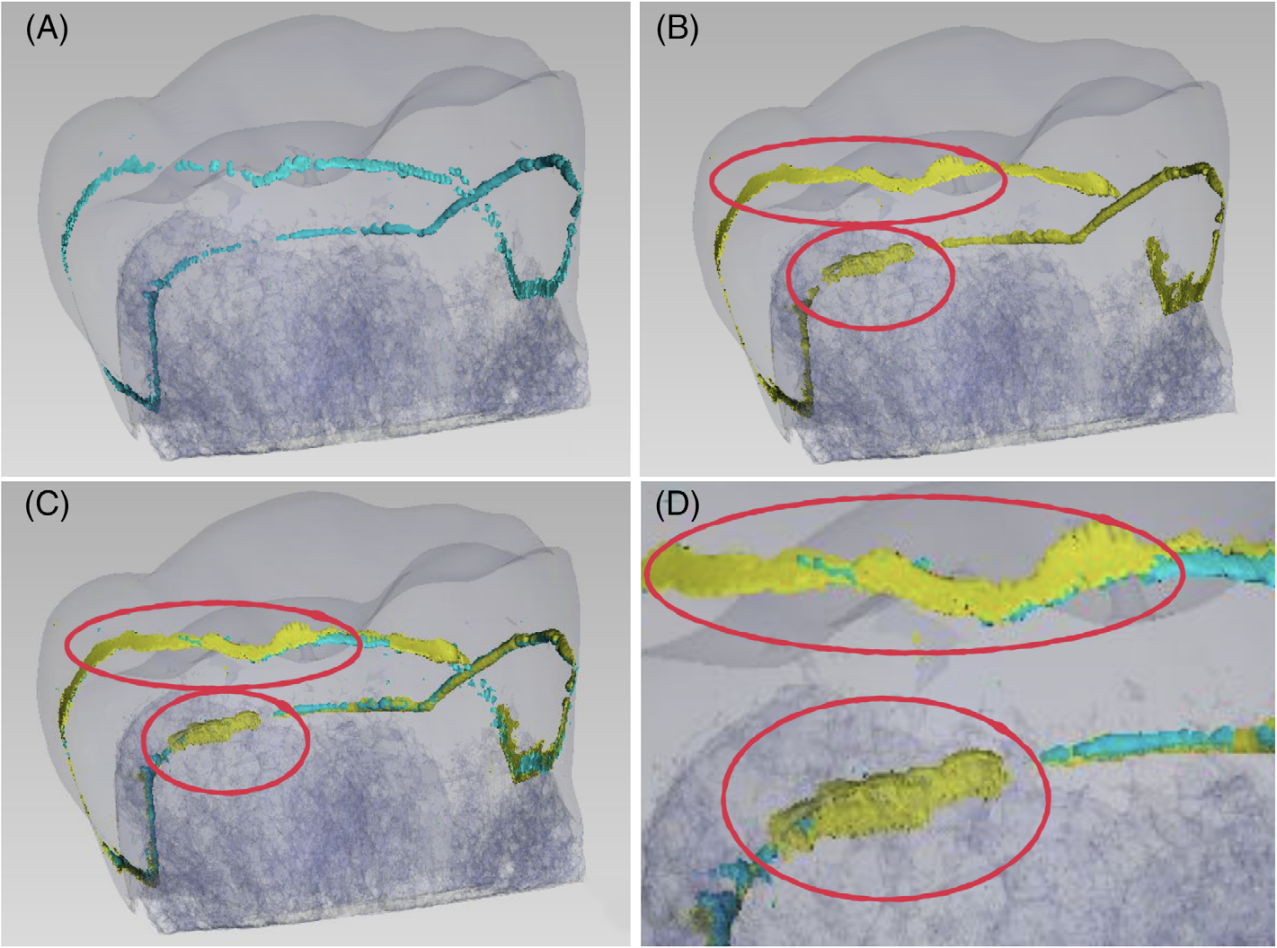
Random sample (ADH, HTZ) external gap progression analysis. Figure A presents the baseline gap in light blue aligned with the transparent blue tooth‐restoration complex. It is worth mentioning that even if a gap is reported throughout the whole interface, it is extremely thin, making its total volume almost irrelevant. Figure (B) presents the same sample gap after fatigue simulation in yellow, with red circles indicating some of the area that showed a significant gap progression. Figure (C) presents the superimposition of the baseline (light blue) on the final gap (yellow), with the same highlights presented in Figure B. Figure (D) presents a detailed view of Figure C for better understanding

To have significant data to discuss and to highlight the interfacial gap progression caused by cyclic fatigue, a subtraction was made between the final gap volume and the baseline gap volume. Figure 3 presents a random sample gap analysis, before and after the chewing simulation, with the external gap progression highlighted.

### 2.5 Statistical analysis

A Shapiro–Wilk test revealed that the data were normally distributed. To evaluate the effect of materials and preparation design on the tridimensional interfacial gap progression, a two‐way analysis of variance (ANOVA) and post‐hoc Tukey test were performed. The significance level was set to 95% (*p* < 0.05). All statistical analyses were performed using the STATA software package (ver. 14.0, StataCorp, College Station).

## 3 RESULTS

None of the tested specimens showed critical cracks, fractures, or debonding after cyclic fatigue. The external gap progression data (±*SD*, expressed in cubic millimeters) of the tested specimens are shown in Table 3.

**TABLE 3:** External gap progression, expressed as mean ± standard deviation (mm^3^) for all tested subgroups

	HTZ	ZLS
ADH	0.16 ± 0.08	0.10 ± 0.06
CRW	0.11 ± 0.09	0.02 ± 0.02

Two‐way ANOVA reported significant differences between the tested materials (*p* = 0.0001) and preparation designs (*p* = 0.005), while their interaction did not show a significant difference (*p* = 0.75). Pairwise comparison showed that the high‐retentive preparation design had a significantly inferior gap progression compared to the overlay preparation. However, ZLS exhibited an inferior gap progression compared to HTZ.

## 4 DISCUSSION

Degradation of restorative interfaces is a key topic in better understanding and preventing biomechanical and microbiological failures of modern restorations that use adhesion to properly reinforce tooth structures while preserving dental tissues.^4^, ^29^ In the present study, cyclic fatigue simulation induced external gap progression in all specimens, in agreement with several papers that previously demonstrated that fatigue stresses are able to cause degradation of adhesive interfaces.^30^, ^31^ Although there is no clear correlation between in vitro gap formation and interfacial failures observed in vivo, none of the specimens showed external gap higher than 150 μm after cyclic fatigue, which is considered clinically acceptable.^32^

Based on the present study's results, the first null hypothesis was rejected, since the high‐retentive preparation design showed lower external gap progression than the low‐retentive ones. Several explanations might be offered for this finding. First, adhesive cementation helps to distribute forces, ultimately improving a restoration's fatigue resistance.^33^, ^34^ The tested high‐retentive design possessed a wider adhesive interface, which might have acted as a cushion, better dissipating forces and preventing gap progression. Second, the fatigue simulation included a sliding movement meant to increase the lateral forces applied to the restorative material, forcing the system to flex. Therefore, the axial walls of the crown design probably dissipated some of these lateral forces, acting like a ferrule and increasing not only the retention but also the stability of the system.^35^, ^36^ Finally, gap progression in low‐retentive restorations was probably augmented due to the direction of the chewing sliding pattern, which started from the central fossa and moved along the buccal triangular crest. In fact, in the selected adhesive overlay design, buccal and oral cusps had the lowest stability due to the lack of vertical walls. Moreover, the different margin configuration and the consequent restoration marginal profile could also justify the external gap progression showed in the present study. In fact, the beveled chamfer of the low‐retentive preparation corresponds to a wider amount of enamel exposure but a slightly thinner restoration in the external part, that might be more prone to chipping.^37^ Partially in disagreement with the present study's results, a recent paper by Gupta et al. reported that both zirconia crowns and overlays had similar marginal behavior after fatigue.^38^ However, they performed their analysis with SEM and focused on microcracks and marginal integrity rather than volumetrically quantified gaps; thus, it is impossible to directly compare results. As underlined in a review on marginal adaptation, micro‐CT is the only method that allows both a precise identification of critical gaps and sufficient measurements to define margin conditions.^39^

The results of the present study also showed significant differences between ZLS and HTZ in terms of gap progression; thus, the second null hypothesis was rejected. A first possible explanation regarding the external gap progression results concerns the adhesive cementation. In fact, it has already been demonstrated that ZLS can be successfully luted, achieving high bond strength, if the surface is properly treated.^40^ However, HTZ, due to the absence of any glassy matrix, cannot be conditioned with conventional acid etching techniques and, consequently, might be considered less suitable for adhesive procedures.^41^, ^42^ Thus, the stability of the HTZ cement‐restoration interface might be inferior compared to that of ZLS. A recent study on tensile bond strength in ZLS showed good performance and stability with aging, even if thermocycling significantly influenced the bond strength values.^43^ Similar studies on zirconia, however, reported major loss of bond strength after thermocycling, with prevalent adhesive failures, even if data were cement‐dependent.^44^, ^45^ Another possible explanation relates to the mechanical proprieties of HTZ compared to those of ZLS. HTZ has a flexural strength of approximately 600–800 MPa and an elastic modulus of 200–210 GPa,^11^ while ZLS flexural strength of 400–500 MPa and an elastic modulus of 60–67 GPa.^46^ Several papers support the fact that low elastic modulus restorative materials have better biomechanical performance when applied to full‐coverage adhesive restorations. They demonstrate a better stress distribution due to the partial absorption of stress^47^, ^48^ which might cause interfacial overloading in HTZ samples, ultimately bringing to premature fatigue micro‐failure of the restorative interface, recorded as volumetric gap progression in the present study's methodology.

Within the limitations of the present study, it is worth mentioning the difficulty encountered in HTZ sample analysis due to the presence of X‐ray artifacts. Micro‐CT has been widely used to analyze the internal and marginal fit of zirconia crowns^49^, ^50^ and therefore can be considered a reliable method of qualitative analysis. However, when it comes to quantitative evaluation through software‐automated analysis, thresholding of gap was found to be harder in HTZ than in ZLS. The scattering effect of HTZ caused pixel blurring that the software sometimes incorrectly included in the region of interest. This problem was managed with a few manual adjustments and a modification of the acquisition phase, as described in Section 2.

## 5 CONCLUSIONS

Based on the obtained results and within the limitations of the present study, it can be concluded that external gap progression was significantly inferior for the high‐retentive preparation design and significantly lower for ZLS compared to HTZ.

Further studies are necessary to confirm the given results, to provide a better understanding of the biomechanical behavior of HTZ and ZLS in minimally invasive dentistry and to find a possible correlation between the marginal gap progression and the interfacial bacterial colonization in indirect adhesive restorations.

## AUTHOR CONTRIBUTION

All authors have contributed significantly and are in agreement with this article.

## Data Availability

The data that support the findings of this study are available from the corresponding author upon reasonable request.
